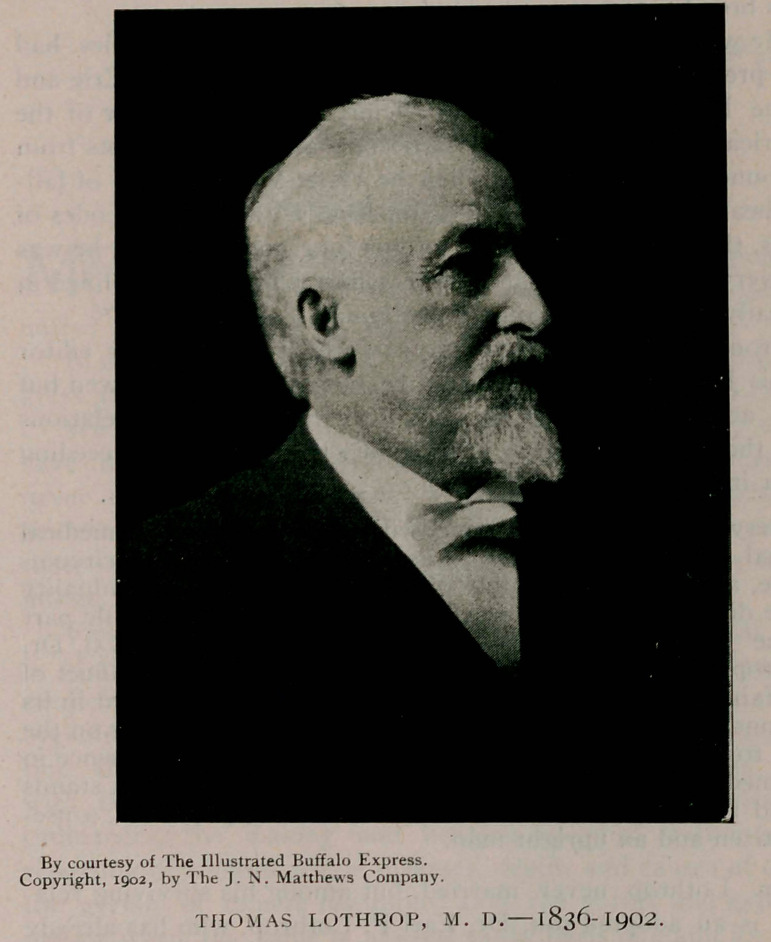# Thomas Lothrop, M. D.

**Published:** 1902-09

**Authors:** 


**A Monthly Review of Medicine and Surgery.**

*EDITOR:*

WILLIAM WARREN POTTER, M. D.

All communications, whether of a literary or business nature, books for review and exchanges should be addressed to the editor: 284 FRANKLIN STREET, BUFFALO, N. Y.

VOL. XLII.—LVIII. SEPTEMBER, 1902. NO. 2.

### Thomas Lothrop, M. D.

AT RARE intervals the JOURNAL, in fulfilment of its solemn obligations, is called upon to chronicle the death of a former or present member of its editorial corps. In this instance there is special sadness attached to this duty, because Dr. Lothrop was no ordinary man, no commonplace citizen, no undistinguished physician. He was indeed the very opposite of all these. He was not an ordinary man for his physical organisation and mental powers were unusually developed; he was no commonplace citizen, because he took an active part in civic, educational and social development; he was not undistinguished as a physician, because he attained unusual eminence in his profession and had the respect of his colleagues far beyond the average. In all of these capacities he enjoyed the trials and triumphs incident to a busy and well-ordered life.

Thomas Lothrop was born in Provincetown, Mass., April 16, 1836, and died at Buffalo, August 7, 1902, and was therefore 66 years, 4 months and 22 days old at his decease. His early education was received at private schools and at Clinton Liberal Institute, and he graduated in medicine at the University of Michigan in 1858. He soon established himself in practice in Buffalo, locating first at Black Rock, and in 1871, removed to the more central part of the city. From this time his professional career was a most active one, being only partially interrupted by a single term as Superintendent of Public Schools, as the office was then termed, until his death.

In 1883 he founded, in association with Dr. John Cronyn, the Medical Department of Niagara University, and was its acknowledged head for several years before it was united with the University of Buffalo. In the former he was professor, and in the latter honorary professor, of obstetrics. As a teacher he achieved conspicuous honor, and was a master in his special

By courtesy of The Illustrated Buffalo Express.
Copyright, 1902, by The J. N. Matthews Company.

THOMAS LOTHROP, M. D.—1836-1902.

department. He established the Woman's Hospital, which was first a maternity and afterward a gynecological institution. In this his pupils learned clinical obstetrics and became well equipped on graduation. He has had the usual hospital service as attending and consulting physician in several institutions in this city,—the Sisters of Charity, St. Francis's, St. Mary's Maternity, Providence Retreat, etc., and was appointed in 1892, one of the managers of the Buffalo State Hospital. He was an active member of the board of management of the Church Charity Foundation and succeeded to the presidency thereof upon the death of Dr. James P. White in 1881. He was also president of the board of trustees of the State Normal School at the time of his death. From all this, which is necessarily but a brief sketch, it will be observed that he was truly a man of affairs, in its broad sense.

He was a member of the several local medical societies, had been president of the Medical Society of the County of Erie and of the Buffalo Academy of Medicine, and was a Fellow of the American Association of Obstetricians and Gynecologists from its foundation until 1899, when he resigned on account of failing health. He had small sympathy with written codes of ethics, though in every walk of his long and useful life he was the very apostle of that higher ethics which is exemplified in the daily conduct of a courtly gentleman.

From 1879 to 1899—twenty years—he was the senior editor of this JOURNAL, though for ten years or more he bestowed but little active work upon its columns. He severed his relations with the JOURNAL August 1, 1899, at which time the succeeding editor-in-chief wrote:

> Very few men have sustained editorial relations to a medical journal for so long a period as twenty years and this circumstance, alone, speaks strongly of the character and individuality of the distinguished retiring editor. During a considerable part of the time he maintained relationship to the JOURNAL Dr. Lothrop was an active, if not a leading spirit in the conduct of its affairs. His ready and forceful pen was often wielded in its columns and his tactful business acumen guided its career on the road to success. He has occupied a position of prominence in the medical profession during all this time and today stands second to none in all that constitutes a strong physician, a useful citizen and an upright man.

Dr. Lothrop never married, but among his surviving relatives is an adopted son, Dr. Earl P. Lothrop, who has already taken a prominent position in the profession and succeeds to a large part of the estate of his foster-father. The others who survive are three sisters and a brother: Mrs. Adeline Nickerson, of Somerville, Mass.; Mrs. Rebecca Fielding and Mrs. Rotie Cook, both of Provincetown, Mass.; and Dr. Benjamin Lothrop of this city.

Such a career of usefulness and honor is vouchsafed to only a few men, and Dr. Lothrop left the precious heritage of a spotless life to the municipality in which he lived and moved so long, to the profession which he adorned so much, and to his surviving kin whom he loved so well. As a final thought we beg to offer our sympathy to those who survive, in which we feel sure the entire profession of medicine in this vicinity will join.

## Figures and Tables

**Figure f1:**